# Treatment approaches in advanced penile cancer: targeted therapies and immunotherapy

**DOI:** 10.3389/fonc.2024.1457006

**Published:** 2025-01-15

**Authors:** David J. Benjamin, Robert C. Hsu

**Affiliations:** ^1^ Department of Medical Oncology, Hoag Family Cancer Institute, Newport Beach, CA, United States; ^2^ Department of Internal Medicine, Division of Medical Oncology, Norris Comprehensive Cancer Center, University of Southern California, Los Angeles, CA, United States

**Keywords:** penile cancer, targeted therapy, immune checkpoint inhibitor, antibody drug conjugate, vaccine

## Abstract

Penile cancer is a rare genitourinary malignancy which can be treated with surgery or radiation for localized disease, but often requires systemic treatment with chemotherapy for recurrent or metastatic disease. With the emergence of immune checkpoint inhibitors and targeted therapies for specific genomic aberrations in the treatment of over a dozen other cancers, recent studies have sought to identify therapies other than chemotherapy in treating this uncommon cancer. Several ongoing trials involving immune checkpoint inhibitors, tyrosine kinase inhibitors, and antibody drug conjugates are attempting to identify additional therapies.

## Introduction

Penile cancer is an uncommon genitourinary malignancy that accounts for less than 1% of male cancers in the United States (1). Utilizing the GLOBOCAN database, the estimated incidence rate of penile cancer globally was 0.80 per 100,000 and estimated mortality rate was 0.29 per 100,000 in the year 2020 (2). In other words, there were an estimated 36,068 new cases of penile cancer and 13,211 deaths from penile cancer that year alone. Several factors for the development of penile cancer have been identified including but not limited to smoking, phimosis, obesity, human papilloma virus (HPV), lack of circumcision, and poor personal hygiene (3).

Disparities in incidence based on region of diagnosis have been identified, with higher incidence rates observed in South Asia, Southern Africa, and South America. In fact, the highest incidence rates per 100,000 were estimated to be in the countries of Eswatini (7.0), Uganda (4.6), and Botswana (4.4) (2). Similarly, the highest mortality rates were experienced by Eswatini (3.5 per 100,000) and Uganda (2.4 per 100,000. Indeed, low- and middle-countries appear to shoulder nearly twice the incidence and mortality compared to high-income countries. It is theorized that infectious diseases including HIV and HPV as well as circumcision patterns may explain the differences in incidence of this rare genitourinary malignancy between different regions. Nonetheless, the incidence of penile cancer appears to be increasing in several regions of the world and warrants further attention given its growing burden to healthcare systems globally.

## Staging

Penile cancer is generally staged using the American Joint Commission on Cancer staging system (8^th^ edition, 2017) incorporating tumor (T), regional lymph nodes (N) and distant metastasis (M) (4, 5). T1 tumors are defined as glans tumors invading the lamina propria, foreskin tumors invading the dermis, lamina propria or dartos fascia, shaft tumors invading connective tissue between the epidermis and corpora, or any site with or without lymphovascular invasion or perineural invasion. T2 tumors are defined as those invading into the corpus spongiosum (either the glands or ventral shaft) with or without the presence of urethral invasion. T3 tumors include those with invasion into the corpora cavernosum including the tunica albuginea with or without the presence of urethral invasion. Finally, T4 tumors invade adjacent pelvic structures such as the prostate, scrotum or pubic bone.

The presence of regional lymph node involvement is critical in the treatment approach of penile cancer. Individuals without palpable or visibly enlarged inguinal lymph nodes are categorized as having cN0 disease, while those with palpable mobile unilateral inguinal lymph nodes are categorized as having cN1 disease. In addition, cN2 disease is defined as the presence of 2 or more palpable mobile unilateral inguinal lymph nodes or bilateral inguinal lymph nodes while cN3 disease is defined as having either unilateral or bilateral fixed inguinal nodes or pelvic lymph nodes.

## Treatment

The treatment approach for penile cancer varies depending on staging, particularly with lymph node involvement or the presence of metastatic disease. In men who have localized T1 disease with low grade tumors may be treated with surgery including local excision, partial penectomy, laser therapy, or radiation therapy. However, men with T2 or greater tumors may be treated with either partial or total penectomy or radiation therapy with or without chemotherapy. For those with low-risk disease, non-palpable inguinal lymph nodes may be monitored with surveillance while in those with T1b or greater disease, non-palpable inguinal lymph nodes may be assessed with bilateral dynamic sentinel node biopsy (DSNB) or treated with bilateral inguinal lymph node dissection (ILND) followed by surveillance. The presence of palpable inguinal or pelvic lymph nodes may warrant percutaneous biopsy and may be treated with either pelvic lymph node dissection (PLND), radiation therapy with or without chemotherapy, or may warrant neoadjuvant chemotherapy such as TIP (paclitaxel, ifosfamide and cisplatin). The detailed management of lymph node positive penile cancer is further described in a recent systemic review by Sachdeva and colleagues (6).

Per the National Comprehensive Cancer Network (NCCN) Guidelines (Version 1.2024), the preferred first-line systemic therapy for metastatic or recurrent penile cancer is TIP. Of note, no randomized controlled trial has been performed in men who had distant metastatic disease as the data for TIP originates from a phase II study including men who had either stage N2 or N3 disease. In the trial, thirty men received four cycles of TIP and had a 50% objective response (7). With a median follow-up of 34 months, the trial found the median time to progression was 8.1 months (95% CI, 5.4 to 50+) while the overall survival was 17.1 months (95% CI, 10.3 to 60+). Among the 30 patients, 20 had died with seventeen of these deaths being attributed to progression of metastatic penile cancer. The other recommended first-line treatment option for metastatic/recurrent penile cancer is 5-fluorouracil (5-FU) in combination cisplatin. The rationale for this combination therapy is based off a pilot study including a total of 8 patients with advanced squamous cell carcinoma of the penis, defined as having either Jackson stages III or IV disease. The study found that 2 (25%) patients had a partial response (8). Given the evidence behind the first-line systemic therapies currently employed in the treatment of metastatic/recurrent penile cancer, we performed a review of the literature to evaluate other systemic therapeutic approaches particularly with the advent of targeted therapies based off genomic aberrations as well as immune checkpoint inhibitors.

## Genomic profiling

Next generation sequencing has allowed for the rapid identification of potentially actionable genomic alterations in tumor samples. A recent systematic review including 7 studies involving 268 cases of penile squamous cell carcinoma identified *TP53*, *CDKN2A*, *FAT1*, *NOTCH-1* and *PIK3CA* as the most frequently occurring mutations (9). In addition, the study identified alterations in *EGFR*, which has several corresponding approved therapies in other malignancies such as head and neck cancer as well as non-small cell lung cancer. In one of the largest genomic profile studies to date, 108 samples of penile squamous cell carcinoma were evaluated. The most common mutations identified in the study were as follows in descending order of frequency: *TP53* (45.5%), *CDKN2A* (25.6%), *PIK3CA* (24.8%), *TERT* (22.2%), *KMT2C* (15.9%), *NOTCH1* (14.1%), *KMT2D* (13.6%), *FBXW7* (8.8%), *NFE2L2* (7.1%), *FAT1* (6.9%), *NF1* (6.5%), *CREBBP* (4.4%), and *FGFR3* (4.3%) (10). In addition, the study found that 10.7% of tumors had a high tumor mutational burden (TMB) defined as 10 or greater mutations per megabase (Mb), and that 1.1% were microsatellite-high (MSI-H) and as such, may be more responsive to treatment with immune checkpoint inhibitors.

A study of 397 patients with penile squamous cell carcinoma found that 15% of cases had a TMB of 10 mut/Mb or greater, with 4% of tumor specimens harboring a TMB of 20 mut/mb or greater (11). A separate study of 72 cases of penile cancer found PD-L1 expression in 79% of cases, thus further suggestive of the potential responsiveness and rationale for utilizing immune checkpoint inhibitors in treating penile cancer (12). Given genomic profiling suggesting possible clinical benefit, several case series and prospective studies have evaluated the efficacy of targeted therapies and immune checkpoint inhibitors in advanced penile cancer.

A separate retrospective study of patients with penile cancer evaluated the findings of germline testing among 29 patients. The study found that 3 patients (10.3%) harbored pathogenic germline variants including 2 with *BRCA2* mutations and one with *RAD51C* (13). In addition, 16 patients had variants of unknown significance. The study is the first and only known evaluation of the germline mutations in patients with penile cancer, as well as the only known study to identify potentially clinically actionable mutations in those with *BRCA2* mutations.

## Immunotherapy

It is estimated that approximately 40% of individuals with cancer in the United States may be eligible for treatment with immune checkpoint inhibitors, which utilize the immune system to kill cancer (14). Given the paucity of data in the treatment of advanced penile cancer, several studies have been undertaken to evaluate the safety and efficacy of immunotherapy in treating this rare malignancy. The first known study to evaluate immune checkpoint inhibitors in advanced penile squamous cell carcinoma involved a patient who was treated with nivolumab after experiencing progression of cancer following chemotherapy and radiation. Treatment with nivolumab led to a partial response with tumor shrinkage, and as such, demonstrated potential efficacy in this patient population (15). A subsequent publication involving two men who had partial penectomy and a combination of chemotherapy and radiation either in the neoadjuvant or adjuvant settings evaluated the efficacy of the immune checkpoint inhibitor pembrolizumab. Both patients’ tumor samples were suggestive of responsiveness to immunotherapy, with one tumor demonstrating a high TMB of 14 mutations/Mb and the second tumor demonstrating positive PD-L1 expression (16). In both instances, patients had a clinical response with the former patient experiencing a complete response for at least 38 months and the latter patient experiencing a partial response for at least 18 months. A separate case series from Hahn and colleagues evaluated three individuals with recurrent, locally advanced or metastatic penile cancer who had experienced progression of cancer on platinum-based chemotherapy. The data from the case series originated from a phase II basket trial for rare malignancies. All three individuals were treated with pembrolizumab, with one patient experiencing a partial response and underwent additional surgery while the two other individuals experiencing progression of their cancer within 3 months of treatment with pembrolizumab (17). Of note, none of the patients experienced grade 3 or worse treatment-related adverse events, thus highlighting the potential safety of this treatment regimen. Finally, the immune checkpoint inhibitor cemiplimab was demonstrated to lead to a complete response in an elderly male with metastatic penile cancer (18). Based off data from these case series and pre-clinical data suggesting potential efficacy for immune checkpoint inhibitors, a limited number of prospective clinical trials have been performed to evaluate the efficacy of immunotherapy in penile cancer.

A multi-center, single-arm phase 2 study evaluated nivolumab plus ipilimumab in patients with advanced rare genitourinary cancers including penile cancer (19). Among the total 55 patients enrolled in the trial, none of the individuals with penile cancer demonstrated a clinical response to treatment with either a partial or complete response. The best response noted among individuals with penile cancer was 2 individuals who experienced stable disease, while 3 experienced progression of their disease.

The first phase II trial evaluating platinum-based chemotherapy plus an immune checkpoint inhibitor was the HERCULES (LACOG 0218) trial (20). In the single arm study conducted at eleven centers in Brazil, a total of 37 patients were enrolled to receive platinum-based chemotherapy plus pembrolizumab for either locally advanced, recurrent or metastatic disease. Of the 33 patients eligible for efficacy analysis, the objective response rate was 39.4% with 1 complete response and 12 partial responses. The response rate was 75% (3 of 4) in patients whose tumors harbored high tumor mutational burden (TMB) status. Moreover, HPV16 positive tumors had a higher response rate (55.6%) in comparison to tumors that were not (35.0%). With a median follow-up of 24.0 months, the median PFS was 5.4 months (95% CI, 2.7-7.2) and median OS was 9.6 months (95% CI, 6.4-13.1). The trial also offered further insight into the most common genomic alterations in the cohort including TP53 (57.1%), CDKN21 (51.4%), and TERT (31.4%).

Despite two positive prospective phase II trials evaluating the use of immunotherapy, the phase II PERICLES trial failed to meet its primary objective of 1-year PFS for the cohort. The study evaluated the efficacy of immune checkpoint inhibitor atezolizumab in 32 patients with stage IVa penile cancer (21). The trial consisted of two cohorts, with cohort A receiving atezolizumab in combination with radiation therapy for those with locoregional disease, and atezolizumab monotherapy in cohort B. The trial found a one-year PFS of 12%, with a median OS of 12 months. Of note, a complete response was noted in two patients with pulmonary metastases. A summary of all clinical trials and case series evaluating the safety and efficacy of immune checkpoint inhibitors is available in **Table 1**.

**Table 1:** Prospective studies and case series utilizing immune checkpoint inhibitors in treating penile cancer.

Patient Population	Study Type	Immune Checkpoint Inhibitor	Clinical Response	Citation
Locally advanced, recurrent or metastatic penile squamous cell carcinoma	Single arm, phase II trial	Pembrolizumab (in combination with platinum-based chemotherapy)	1 complete response, and 12 partial responses. Objective response rate of 39.4%. Median PFS was 5.4 months and median OS was 9.6 months	Maluf FC, et al. (20) Journal of Clinical Oncology. 2024.
Rare genitourinary cancers including penile cancer	Single arm, phase II multi-center trial	Nivolumab plus ipilimumab	No partial or complete response. 2 individuals with stable disease, and 3 with disease progression	McGregor BA, et al. (19) Cancer. 2021.
Stage IVa penile cancer	Phase II, single-center trial	Atezolizumab (with or without radiotherapy)	Two complete responses in patients with pulmonary metastases. One year PFS of 12%. Median OS was 12 months	de Vries HM, et al. (21) Journal of Clinical Oncology. 2022.
Advanced penile squamous cell carcinoma refractory to chemotherapy plus radiation	Case Report	Nivolumab	Partial response	Trafalis DT, et al. (15) Journal of Immunotherapy. 2018.
Two males refractory to partial penectomy and chemotherapy and radiation in either neoadjuvant or adjuvant settings	Case Report	Pembrolizumab	1 complete response, 1 partial response	Chahoud J, et al. (16) Frontiers in Oncology. 2020.
Three patients with recurrent, locally advanced or metastatic penile squamous cell carcinoma progressed on platinum chemotherapy	Case series from Phase II basket trial	Pembrolizumab	1 partial response, 2 patients experienced progression of disease	Hahn AW, et al. (17) Investigational New Drugs. 2021.
Metastatic squamous cell carcinoma of the penis	Case report	Cemiplimab	Complete response	Denis C, et al. (18) Case Reports in Oncology. 2021.

## Targeted therapies

Given genomic profiling demonstrating the presence of *EGFR* alterations, *EGFR*-directed therapies have been utilizing in treatment recurrent or advanced penile cancer. In one of the first case series, 24 patients with advanced penile or scrotal cancer received *EGFR*-directed therapies including cetuximab, erlotinib and gefitinib (22). Of these patients, 20% (1/5) had a partial response seen on imaging with cetuximab alone, while 25% (3/12) who received cisplatin in combination with cetuximab had partial responses. No individuals treated with gefitinib or erlotinib demonstrated clinical benefit to *EGFR*-targeted treatment. A subsequent case series evaluated treatment outcomes with *EGFR*-directed therapies in 3 patients with advanced penile cancer whose cancers were refractory to chemotherapy. Of the three patients, one patient had a complete response to cetuximab and remained disease-free for 42 months while the second patient initially responded to panitumumab before experiencing disease progression (23). The third patient did not experience clinical benefit from *EGFR* directed therapy, and experienced disease progression.

A case report of metastatic penile cancer in China reported several potentially actionable findings on genomic testing, including a TMB of 13.97 mutations/Mb as well as *BRCA2* mutation (24). After experiencing radiographic progression on first-line therapy with chemotherapy and immunotherapy, the patient was treated with poly ADP-ribose polymerase (PARP) inhibitor olaparib in the second line setting and experienced a clinical benefit lasting for 9 months. Of note, the patient was also treated with nivolumab plus ipilimumab in the third line setting but had a PFS of 3 months, thus demonstrating a lack of clinical benefit with dual immune checkpoint inhibition despite genomic profiling revealing TMB high status.

The *NTRK* inhibitor entrectinib has been studied in several prospective trials in treating locally advanced or metastatic *NTRK* fusion positive solid tumors. In pooled analysis from three phase 1 and 2 trials, one patient with penile cancer was included in the trial cohort (25). However, tumor response was not specifically reported among tumor types with four or less patients. To our knowledge, no other reports of utilizing targeted therapies based off genomic alterations have been published in the literature thus far.

## Ongoing trials and future directions

Several prospective trials are evaluating the efficacy of immune checkpoint inhibitors in treating penile cancer including a study combining dostarlimab with PARP inhibitor niraparib (ClinicalTrials.gov ID: NCT05526989). In addition, an ongoing phase 2 trial in men who are unfit or had disease progression on platinum chemotherapy is evaluating the efficacy of immune checkpoint inhibitor avelumab (NCT03391479). Avelumab is also being evaluated as maintenance therapy in patients with locally advanced and metastatic squamous cell penile cancer who had treatment response to chemotherapy (NCT03774901). The EPIC trial is an ongoing phase 2 study in the United Kingdom evaluating the efficacy of cemiplimab as monotherapy or in combination with standard-of-care chemotherapy in locally advanced or metastatic penile cancer (26).

Given overexpression of the vascular growth factor (VEGF) receptor in penile cancer, the tyrosine kinase inhibitor cabozantinib is being further studied in this malignancy (27). CaboPen is a single-arm, single-center, phase 2 trial with patients with either locally advanced penile squamous cell carcinoma or in those with metastatic disease, cabozantinib is being studied until disease progression or unacceptable toxicity (NCT03943602). Cabozantinib is also being studied in combination with nivolumab with or without CTLA-4 inhibitor ipilimumab in patients with metastatic genitourinary tumors (NCT02496208), and in a separate trial combining all three aforementioned therapies in rare genitourinary cancers such as penile cancer (NCT03866382).

As HPV-positive and -negative have distinct molecular features and tumor microenvironments, HPV-directed therapies such as vaccines offer a potential opportunity for prevention or treatment (28–30). However, to our knowledge, no ongoing trials are evaluating HPV-directed therapies for advanced penile cancer.

Antibody drug conjugates (ADCs) such as enfortumab vedotin, which targets nectin-4, and sacituzumab govitecan, which is directed towards Trop-2 expressing cells, are currently approved in the treatment of metastatic urothelial carcinoma. Given the theoretical capability to deliver chemotherapy specifically towards cancer cells expressing these proteins, there has been interest in exploring the effectiveness of these therapies in penile cancer (31). A phase II study of rare genitourinary cancers is currently enrolling and evaluating the use of sacituzumab govitecan with or without the immune checkpoint inhibitor atezolizumab (NCT06161532). In addition, enfortumab vedotin is also being studied in a phase 2 trial in individuals with unresectable or metastatic squamous cell carcinoma of the penis (NCT06104618). These ADCs as well as others in clinical development represent a promising, more targeted approach in delivering cytotoxic payloads to cancer cells (32). A summary of known ongoing and planned prospective clinical trials in advanced penile cancer is also available in **Table 2**.

**Table 2:** Ongoing and planned prospective clinical trials in advanced penile cancer.

Patient Population	Study Type	Intervention	ClinicalTrials.gov Identifier
Advanced relapsed/refractory penile cancer following chemotherapy	Phase 2	Dostarlimab plus Niraparib	NCT05526989
Locally advanced or metastatic penile cancer who are unfit or progressed on platinum-based chemotherapy	Phase 2	Avelumab	NCT03391479
Maintenance immunotherapy after first-line platinum-based chemotherapy in locally advanced or metastatic squamous cell penile carcinoma	Phase 2	Maintenance avelumab	NCT03774901
Locally advanced or metastatic penile carcinoma	Phase 2	Cemiplimab alone or in combination with standard of care chemotherapy	Not available
Advanced penile squamous cell carcinoma	Phase 2	Cabozantinib	NCT03943602
Metastatic genitourinary tumors	Phase 1	Cabozantinib and nivolumab with or without ipilimumab	NCT02496208
Rare genitourinary tumors in the first or second-line (and beyond) setting	Phase 2	Nivolumab and ipilimumab plus cabozantinib	NCT03866382
Rare genitourinary tumors including small cell, adenocarcinoma, squamous cell bladder/urinary tract cancer, renal medullary carcinoma and penile cancer	Phase 2	Sacituzumab govitecan with or without atezolizumab	NCT06161532
Metastatic or unresectable squamous cell carcinoma of the penis	Phase 2	Enfortumab vedotin	NCT06104618

## Conclusion

Penile cancer remains a challenging malignancy to treat in men particularly in advanced disease. Chemotherapy has historically served as the primary treatment modality in recurrent, locally advanced or metastatic penile cancer. Although several case reports have demonstrated potential clinical efficacy in patients whose tumors harbor *EGFR* or *BRCA2* mutations, prospective data lacks in oncogenic driver mutated penile cancer. Several recent phase II trials have demonstrated clinical benefit in a subset of patients who receive treatment with immune checkpoint inhibitors; however, given the genomic profile of penile cancer, it remains unclear if immunotherapy may benefit most patients with penile cancer. Nonetheless, given the paucity of data for currently employed chemotherapy regimens in these aforementioned settings, several ongoing studies aim to evaluate the safety and efficacy of immune checkpoint inhibitors as well as antibody drug conjugates as potential newer generation approaches in treating this uncommon cancer.
